# Contributions to Operative Surgery and Surgical Pathology

**Published:** 1859-03

**Authors:** 


ART. VII.—*Contributions to Operative Surgery and Surgical Pathology.* By J. M. CARNOCHAN, M.D., Professor of Surgery in the New York Medical College, Surgeon-in-Chief to the State Emigrants' Hospital, etc. With Illustrations drawn from Nature. Part II. 4to., pp. 33–77. Philadelphia: Lindsay & Blakiston. 1858.

THE second part of this work has appeared somewhat later than had been promised by its publishers; it contains an account of a case of exsection of the entire ulna, a case of facial neuralgia, with remarks on this affection, and three cases in which the superior maxillary branch of the fifth pair of nerves was exsected, beyond Meckel's ganglion, for obstinate facial neuralgia.

Much credit is due to American surgeons for their success in exsecting bones in their continuity, and we may justly claim priority in several of the more important of this class of operations. Notwithstanding Dr. Carnochan gives to Dupuytren the credit of having first removed a portion of the inferior maxillary bone, we must insist on the claims of Dr. Deadrick, of Tennessee, who performed the operation three years earlier than the distinguished French surgeon. The entire clavicle was exsected for the first time, in 1813, by Dr. McCreary, of Kentucky, an operation which was performed twelve years later by Dr. Mott, and which, since that time, has been successful in the hands of several American surgeons. In regard to removal of the entire ulna, we would remind the author that he is not, as he seems to suppose, the deviser of the operation, as Dr Robert B. Butt, of Virginia, exsected the whole of the left ulna in 1825, for the same reasons as in the case before us, and with results so happy that the patient was able to resume his occupation as a wood-joiner.

The case of Dr. Carnochan is a particularly interesting one, as it is accompanied with a very full history and description of the operation, and the after appearance and use of the limb. The patient was an Irishman, thirty years of age, who sprained his arm in such a manner as to give rise to severe inflammation from the wrist up to the shoulder-joint. When he entered the State Emigrants' Hospital, the arm was greatly enlarged and indurated, being the seat of exquisite pain, and the integuments were of a purplish color. The constitution sympathized with the local disorder, and the patient was much emaciated. Incisions were made into the parts, followed by the escape of some pus and blood, and appropriate local and constitutional remedies were employed. Abscesses formed over the ulna, giving rise to sinuses, through which dead bone could be discovered. At the expiration of eighteen months the bone was removed; the wound had united in two weeks, and in three weeks more the patient was discharged, cured, from the hospital. The bone was found to be greatly hypertrophied, the seat of caries, necrosis, cloacæ, and eburnation, and weighed nearly eight ounces. These appearances are well shown in two admirable lithographs.

About two years after the operation we had an opportunity of seeing the patient, who presented himself to us on account of a glandular abscess in the right axilla. He was evidently of a strumous habit of constitution. We were struck with the slight deformity and the great usefulness of the limb, all the functions being performed nearly as well as on the sound side.

In the paper on neuralgia of the face, a very remarkable case is related of a man on whom twelve operations had been performed in the space of five years and a half, for severe neuralgia of the second branch of the fifth pair. These consisted in the removal, at various times, of a portion of the nerve, but without any material benefit. The last operation was performed by the author in October, 1857, when about an inch of the nerve was excised. This was followed by an amelioration of the symptoms, but occasional paroxysms afterwards took place. The author states that about one-half an inch of the nerve still remains in front of the ganglion of Meckel, both of which he shall remove if it be deemed necessary.

The third and last paper of this work treats of a novel and bold procedure of the author in intractable cases of neuralgia. This consists in removal of the whole of the second branch of the fifth pair at the foramen rotundum, together with the ganglion of Meckel. The reasons given for the operation may be seen from the following extract:—

"When even a large portion of the trunk of the second branch of the fifth pair has been simply exsected from the infra-orbital canal, the ganglion of Meckel continues to provide, to a great extent, the nervous ramifications, which will still maintain and keep up the diversified neuralgic pains. Besides the ganglion of Meckel, being composed of *gray matter, must play an important part as a generator of nervous power*, of which, like a galvanic battery, it affords a continual supply, while the branches of the ganglion, under the influence of the diseased trunk, serve as conductors of the accumulated morbid nervous sensibility."

Dr. Carnochan adduces three cases treated in this way, all of which have been followed by cures; whether or not the cures have been permanent we are not informed. In all of these cases the nerve was found to be enlarged, vascular, and engorged, appearances which are imperfectly represented in the lithograph.

We look forward with pleasure for the appearance of the next part of this work, which has already proved that its author is an excellent anatomist, a bold operator, and an original thinker.

